# Sedentary lifestyle related exosomal release of Hotair from gluteal-femoral fat promotes intestinal cell proliferation

**DOI:** 10.1038/srep45648

**Published:** 2017-03-31

**Authors:** Xiaozhao Lu, Danna Bai, Xiangwei Liu, Chen Zhou, Guodong Yang

**Affiliations:** 1 Department of Biochemistry and Molecular Biology, Fourth Military Medical University, Xi’an, 710032, China; 2 State Key Laboratory of Cancer Biology, Fourth Military Medical University, Xi’an, 710032, China; 3 The 323rd Hospital, PLA, Xi’an, 710043, China; 4 Department of Physiology, Fourth Military Medical University, Xi’an, 710032, China; 5 State Key Laboratory of Military Stomatology and National Clinical Research Center for Oral Diseases, Fourth Military Medical University, Xi’an, 710032, China; 6 Guanghua School of Stomatology, Hospital of Stomatology, Guangdong Provincial Key Laboratory of Stomatology, Sun Yat-sen University, 56 Lingyuanxi Road, Guangzhou, 510055, China

## Abstract

Pioneering epidemiological work has established strong association of sedentary lifestyle and obesity with the risk of colorectal cancer, while the detailed underlying mechanism remains unknown. Here we show that Hotair (HOX transcript antisense RNA) is a pro-adipogenic long non-coding RNA highly expressed in gluteal-femoral fat over other fat depots. Hotair knockout in adipose tissue results in gluteal-femoral fat defect. Squeeze of the gluteal-femoral fat induces intestinal proliferation in wildtype mice, while not in Hotair knockout mice. Mechanistically, squeeze of the gluteal-femoral fat induces exosomal Hotair secretion mainly by transcriptional upregulation of Hotair via NFκB. And increased exosomal Hotair in turn circulates in the blood and is partially endocytosed by the intestine, finally promoting the stemness and proliferation of intestinal stem/progenitor cells via Wnt activation. Clinically, obese subjects with sedentary lifestyle have much higher exosomal HOTAIR expression in the serum. These findings establish that sedentary lifestyle promotes exosomal Hotair release from the gluteal-femoral fat, which in turn facilitates intestinal stem and/or progenitor proliferation, raising a possible link between sedentary lifestyle with colorectal tumorigenesis.

Intestinal stem cells, which reside at the base of intestinal crypts adjacent to Paneth cells, together with their progeny, are thought as the origin of cancer initiation^1^. Although accumulating evidence from epidemiological studies has link sedentary lifestyle and obesity to colon cancer risk^2^,^3^, little is known about how the changes in the fat affects the distal intestine, especially the stem and progenitor cells there.

Long non-coding RNAs (lncRNAs) are abundantly transcribed in mammalian genomes, some of which act in *trans* to guide silencing complexes to target sites throughout the genome^4^,^5^. For example, HOTAIR (HOX transcript antisense RNA) could recruit the PRC2 and other chromatin modification complexes to the target genes^4^. Accumulating evidences have revealed that lncRNAs are recognized as potent mediators of gene regulation and pathogenic drivers. As a homeobox gene, HOTAIR is transcribed from the syntenic location in the HoxC (homeobox gene cluster C) locus and its expression is restricted in posterior or distal anatomic sites^6^. Accumulating studies implicate that HOTAIR plays an oncogenic role in breast cancer, colorectal cancer and others^4^,^7^,^8^,^9^,^10^. However, the detailed mechanism how HOTAIR is dysregulated in cancer is poorly known. Recently, HOTAIR is found normally and abundantly expressed in the gluteal fat^11^, consistent with its restricted expression in distal anatomic sites. High expression of HOTAIR in the gluteal fat raises the possibility that gluteal fat derived HOTAIR might circulate into the intestine under certain contexts.

Exosomes are endosome-derived vesicles with the diameter range from 40 to 200 nm^12^. Exosomes have been confirmed to be able to transfer the cargoes inside, such as miRNAs, lncRNAs, and proteins, between adjacent or distant cells. Due to the long lasting stability and relatively high target specificity between donor and receptor cells, exosomes nowadays emerge as an essential mediator in intracellular communication especially between distal organs^12^. Recently, adipose tissues are found to be able to secrete exosomal miRNAs and other RNAs and contribute to systemic diseases^13^,^14^. All of these data indicate that gluteal fat derived exosomes might affect the intestinal cell proliferation via HOTAIR inside in the context of sedentary lifestyle.

In this study, we interrogate how sedentary lifestyle influences intestinal stem and progenitor cell function and the underlying mechanisms by focusing on the exosomes secreted from the gluteal-femoral fat depot in a mouse model and clinical samples.

## Results

### Hotair knockout results in gluteal-femoral fat depot specific defect

**Figure 1:**
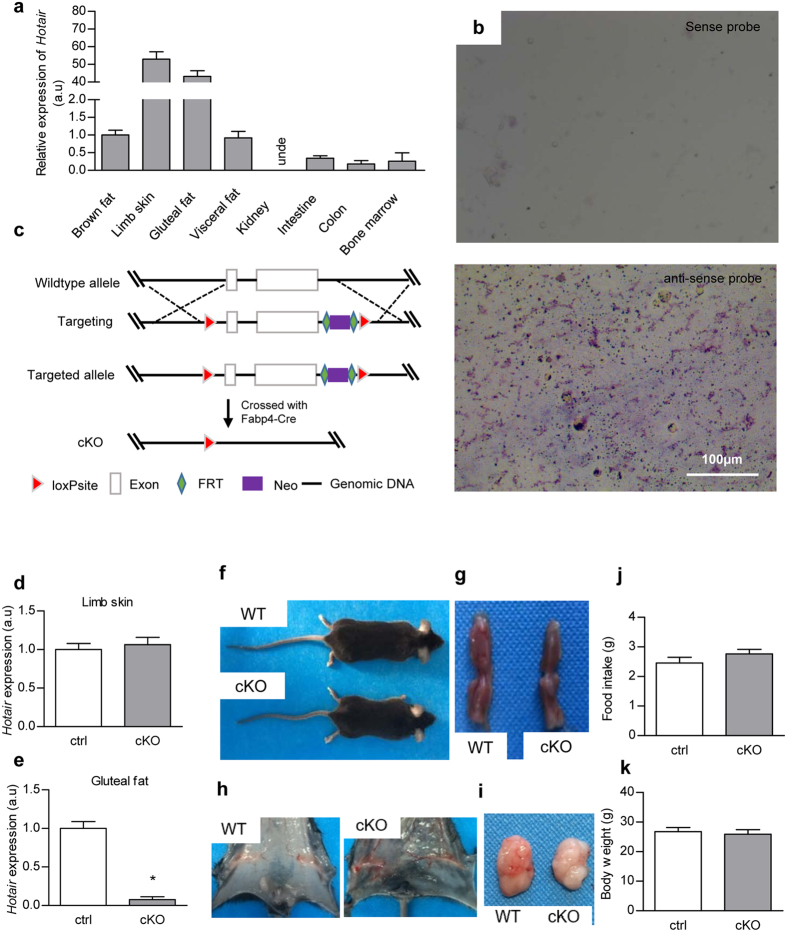
Fat specific deletion of Hotair inhibits gluteal-femoral adipogenesis. (**a**) RT-qPCR analysis of Hotair expression in different tissues in adult mice. Relative expression levels are compared with the expression in brown fats (arbitrary value = 1) and presented as mean ± SEM, three biological replicates with three technical replicates each. (**b**) *In situ* hybridization of Hotair in gluteal fat tissue with sense (upper) and antisense (lower) probes. (**c**) Schematic illustration of the Hotair knockout procedure. (**d**,**e**) RT-qPCR analysis of Hotair in limb skin and gluteal fat. Relative expression levels are compared with the wildtype control (arbitrary value = 1) and presented as mean ± SEM, three biological replicates with three technical replicates each. *Indicates P < 0.05 analyzed by t test. (**f**–**i**) Gross morphology (**f**), brown fat (**g**), gluteal and femoral fat (**h**) and visceral fat (**i**) of control wildtype and fat specific Hotair KO mice. Images presented were representative of five mice. (**j**,**k**) No significant differences were found between control and cKO mice in body weight (**j**) and food intake (**k**). Data presented are representative of at least 3 mice per group.

Previous studies have revealed specific expression of Hotair in the posterior trunk and distal limb bud at embryonic day 11.5 (E11.5), E12.5, and E13.5 stages^6^,^15^,^16^, we here further confirmed the distal limb and posterior trunk expression of Hotair at E16.5 (Supplementary Fig. 1a,b). RT-qPCR analysis of different tissues from the adult mice additionally confirmed its abundant expression specifically in distal limb skin and gluteal-femoral fat depots (Fig. 1a). *In situ* hybridization on the gluteal fat sections further confirmed its high expression in the gluteal fat, while the sense probe had no significant positive signal (Fig. 1b). The gluteal fat site-specific expression pattern of Hotair further supports its specific role in the gluteal fat depot as previously reported^16^,^17^.

To understand the role of Hotair in fat tissue, we thus generated fat specific conditional knockout (KO) mice (hereafter “cKO”) by crossing the Hotair^Loxp/Loxp^ mice with the FABP4-Cre mice (Fig. 1c; Experimental Procedures in methods section). As expected, Hotair (Fig. 1d,e) was specifically deleted in the adipose tissue in Hotair cKO mice. Different from the skeletal abnormalities observed in the ubiquitous Hotair deletion mice^15^, adipose specific deletion of Hotair didn’t cause any obvious skeleton deformation. There were no obvious gross differences neither between wildtype and Hotair cKO mice (Fig. 1f). Detailed examination of the fat depots revealed that there was obvious adipogenic defect in gluteal-femoral fat while not in brown fat in cKO mice (Fig. 1g,h). In addition, the abdominal subcutaneous fat and visceral fat in cKO mice were mildly affected (Fig. 1h,i). Although there was significant adipogenic deficiency in the gluteal-femoral fat, there were no significant differences in food uptake, body weight and blood biochemical parameters between WT and cKO mice (Fig. 1j,k).

### Hotair cKO mice are resistant to “sedentary lifestyle” induced intestinal proliferation

**Figure 2:**
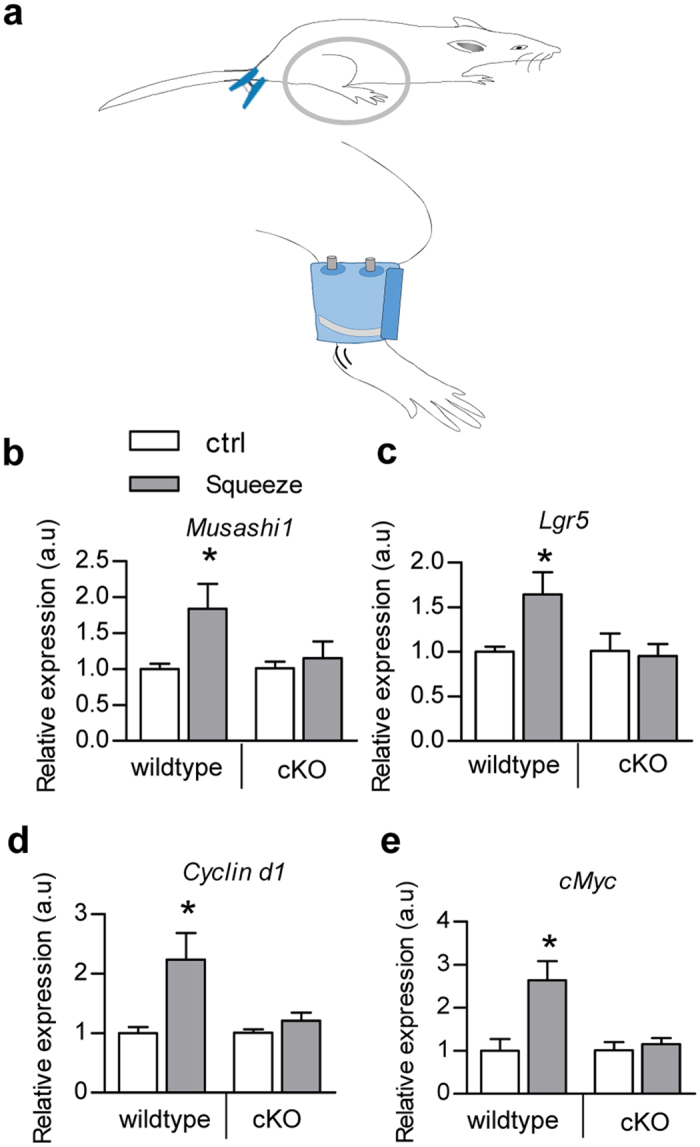
Squeeze promotes intestinal proliferation in wildtype mice, rather than Hotair cKO mice. (**a**) Schematic diagram shows the squeeze procedure how pneumatic cuff applied to the hind limb and clamp of the gluteal region with clip. (**b–e**) RT-qPCR analysis of relative expression of Musashi1 (**b**), Lgr5 (**c**), Cyclin d1 (**d**), and cMyc (**e**) in wildtype and cKO mice with or without squeeze. Relative expression levels are compared with the control (arbitrary value = 1) and presented as mean ± SEM, three biological replicates with three technical replicates each, *indicates P < 0.05 analyzed by one-way ANOVA with Tukey post hoc test.

Epidemiological studies have revealed that sedentary lifestyle is associated with increased colorectal cancer risk, while the detailed mechanism remains obscure. The specific gluteal-femoral fat defect of Hotair cKO mice provides us a rational model to explore the effects of gluteal-femoral fat and fat derived Hotair on the intestinal system. Notably, there were no significant differences in the intestine between control and cKO mice. To further mimic the pressure loaded onto the gluteal-femoral region and insufficient physical activity caused by sedentary lifestyle, a pneumatic cuff was designed for mice and about 20 mmHg pressure was applied to the thigh (Fig. 2a) from midnight to 8:00 am for 3 weeks, left on one day and right the other day. In addition, the gluteal region was clamped for 1 hour every night (Fig. 2a). Compared with the control group, squeeze application significantly increased the intestinal stem/progenitor cell proliferation, as seen from both the stem cell markers and proliferating cell markers in the intestine (Fig. 2b–e). In contrast, the increased intestinal proliferation in intestinal stem/progenitor cells was rarely observed in the Hotair cKO mice (Fig. 2b–e), suggesting either the gluteal-femoral fat or the secreted factors from the fat depot would be mitogenic. Recently, adipose tissue is considered as an endocrine system with diverse roles under different contexts, besides its function in metabolism^18^,^19^,^20^. All of these data suggest that certain circulating factors related to Hotair and/or gluteal-femoral fat might be involved in the squeeze induced intestinal proliferation.

### “Sedentary lifestyle” promotes exosomal Hotair secretion

**Figure 3:**
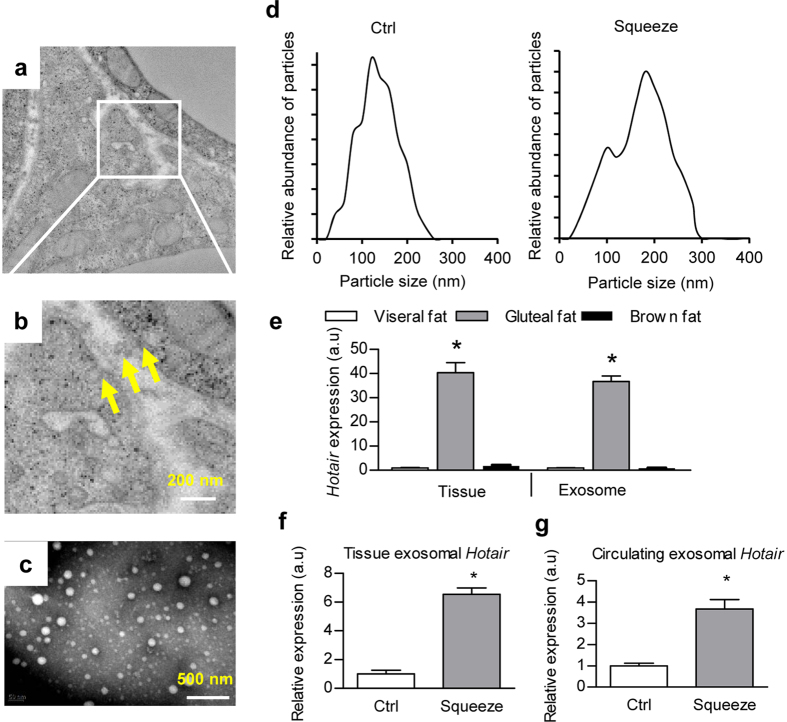
Squeeze induces Hotair expression and exosomal secretion. (**a**) Electron microscope analysis of the gluteal adipose tissue. (**b**) Magnification of the inset in (**a**). (**c**) Electron microscope analysis of the isolated exosomes from gluteal adipose. (**d**) Size distribution of the exosomes from the gluteal fat of control and squeeze treated mice. (**e**) RT-qPCR analysis of cellular and exosomal Hotair expression in different fat depots. Relative expression levels are compared with the visceral fat expression (arbitrary value = 1) and presented as mean ± SEM (three biological replicates with three technical replicates each), *denotes P < 0.05 by one way ANOVA by Tukey’s post hoc test. (**f**,**g**) RT-qPCR analysis of Hotair in gluteal adipose tissue (**f**) and circulating exosomes (**g**) in control and squeezed mice. Relative expression levels are compared with the control (arbitrary value = 1) and presented as mean ± SEM (three biological replicates with three technical replicates each), *denotes P < 0.05 by t test.

Multiple studies have suggested beneficial roles of gluteal-femoral adipose tissue in cardiovascular disease^21^,^22^,^23^,^24^, while its role in colorectal cancer is poorly known. Moreover, Hotair is considered as an oncogenic lncRNA^4^,^7^,^9^. Regarding the capacity of exosomes in transferring functional lncRNAs^12^,^25^, we assumed that Hotair secreted from the gluteal-femoral adipose tissue would probably contribute to the squeeze induced intestinal proliferation. Scanning electron microscope (SEM) analysis of the *in situ* exosomes revealed that the gluteal adipose tissue was active in exosome biogenesis and secretion (Fig. 3a,b). TEM (Transmission electron microscope) and western blot analysis of exosome markers further confirmed the exosome identity (Fig. 3c and Supplementary Fig. 2). Squeeze treatment mildly changed the exosome distribution (Fig. 3d). Consistent with the intracellular expression of Hotair in these different fat depots, there was significantly higher expression of exosomal Hotair in the gluteal fat derived exosomes, when compared with that from abdominal subcutaneous and visceral fat depots (Fig. 3e). Moreover, squeeze treatment increased the exosomal Hotair in both the gluteal fat (Fig. 3f) and the serum (Fig. 3g).

### “Sedentary lifestyle” increases exosomal Hotair via NFκB activation

**Figure 4:**
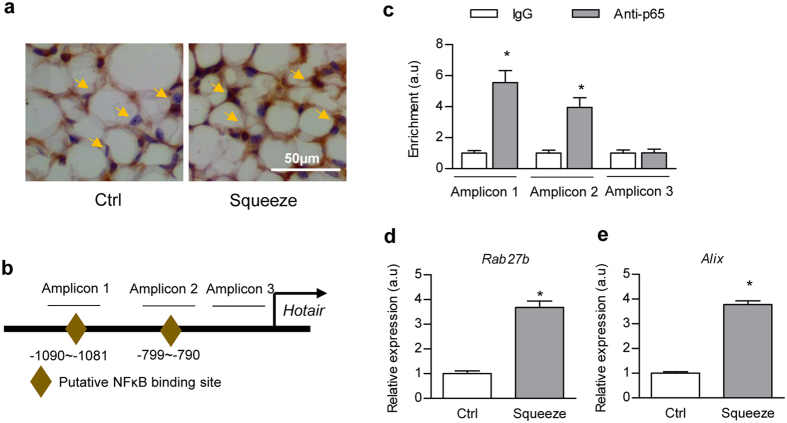
Squeeze enhances exosomal Hotair via NFκB. (**a**) Immunochemistry shows nuclear translocation of NFκB p65 subunit in the gluteal fat upon squeeze. Blue nuclei staining overlapped with brown signal indicates nuclear translocation. (**b**) Schematic illustration of the putative NFκB binding sites in the promoter region of Hotair. (**c**) Chromatin Immunopreciptation (ChIP) analysis of the putative interaction between p65 and the predicted cis-elements on Hotair promoter in the squeezed adipose tissue. Relative enrichment is compared with the IgG control (arbitrary value = 1) and presented as mean ± SEM (three biological replicates with three technical replicates each), *denotes P < 0.05 by One Way ANOVA with Tukey’s post hoc test. (**d**,**e**) RT-qPCR analysis of endogenous expression of exosome biogenesis related Rab27b (**d**) and Alix (**e**) in control and squeeze treated gluteal fat tissue. Relative expression levels are compared with the control (arbitrary value = 1) and presented as mean ± SEM, n = 3, *denotes P < 0.05 by t test.

Next, we explored the mechanism how squeeze promotes exosomal Hotair expression. Immunostaining results revealed that squeeze treatment induced p65 nuclear translocation (Fig. 4a). In addition, bioinformatics analysis suggested multiple NFκB (nuclear factor kappa-light-chain-enhancer of activated B cells) binding sites in the promoter region of Hotair (Fig. 4b), further suggesting possible transcriptional activation of Hotair by NFκB. As expected, ChIP analysis further confirmed the interaction of p65 with Hotair promoter in the squeezed gluteal adipose tissue (Fig. 4c). Consistent with the minor exosome distribution change in squeeze treated gluteal-femoral fat, a few exosome biogenesis related genes, such as Rab27b and Alix, were also found to be upregulated in the squeezed gluteal fat tissue (Fig. 4d,e). Moreover, treatment of *in vitro* cultured ADSCs (Adipose derived stem cells) with TNFα (tumor necrosis factor α), an activator of NFκB, also significantly increased both the cellular and exosomal Hotair expression (Supplementary Fig. 3a,b), with the exosomal Hotair increased much higher. Similarly TNFα treatment also enhanced the expression of Rab27b and Alix (Supplementary Fig. 3c,d), further suggesting additional mechanisms responsible for the increase of exosomal Hotair under NFκB activation. Interaction of p65 with Hotair promoter was also observed in the TNFα treated ADSCs (Supplementary Fig. 3e). Taken together, these data indicate that squeeze treatment, which mimics sedentary lifestyle, increases exosomal Hotair via NFκB activation.

### Circulating exosomal Hotair promotes intestinal cell proliferation

**Figure 5:**
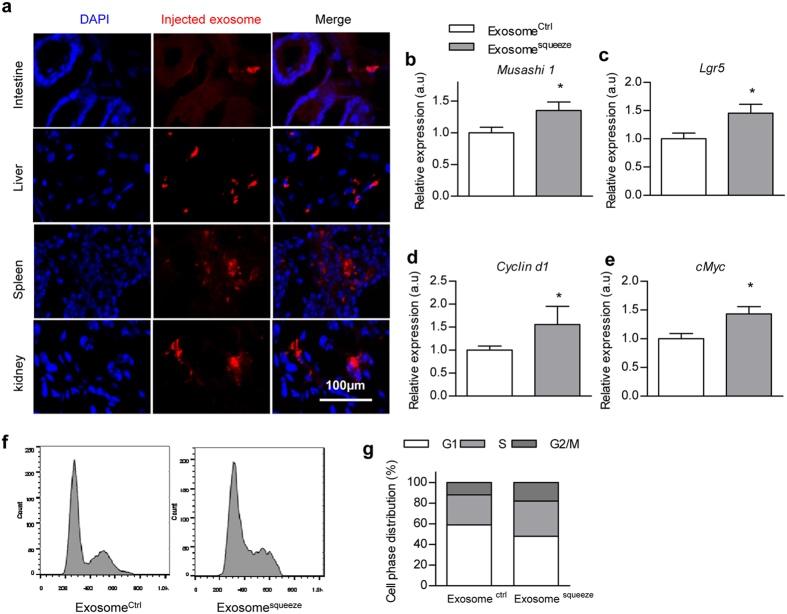
Exosomes from squeezed gluteal fat promote intestinal cell proliferation. (**a**) DiI labeled exosomes were injected via tail vein and *in vivo* distribution of the exosomes were detected by fluorescence microscope. Representative of 3 different experiments. (**b–e**) Mice were treated with exosomes from control or squeezed gluteal fat and intestinal expression of Musashi1 (**b**), Lgr5 (**c**), Cyclin d1 (**d**), and cMyc (**e**) was analyzed by RT-qPCR. Relative expression levels are compared with the control (arbitrary value = 1) and presented as mean 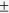 SEM (three biological replicates with three technical replicates each), *P < 0.05 as analyzed by t test. (**f**,**g**) Colon cancer cells were incubated with exosomes from control or squeezed gluteal fat. Cell cycle distribution was analyzed by FACS. Data presented are representative of 3 different experiments.

All of the above data implicate a potential role of exosomal Hotair released from gluteal-femoral fat in promoting intestinal proliferation. We thus tested whether these gluteal-femoral fat derived exosomes could possibly incorporate into the intestine. The isolated exosomes were labeled with fluorescent dye DiI (a lipophilic membrane stain) before tail vein injection. As expected, the labeled exosomes were observed in the intestine besides the liver and spleen (Fig. 5a). Next, the exosomes from the control and squeezed gluteal fat were injected via tail vein respectively. As expected, injection of exosomes from squeezed gluteal fat promoted intestinal cell proliferation, as seen from both the stem cell/progenitor and proliferation markers (Fig. 5b–e), when compared with injection of control exosomes. Moreover, *in vitro* cell culture model study found that transfection of human colon cancer cell line HCT116 with exosomes from the squeezed gluteal fat increased the cell population in S and G2/M phases, as compared with the control (Fig. 5f,g). All of these data indicate that exosomes from squeezed gluteal fat promote intestinal cell proliferation.

To further confirm the role of Hotair in the exosomes in promoting intestinal proliferation, abdominal subcutaneous ADSCs were further infected with control or Hotair expressing virus before exosome isolation. As expected, exosomal Hotair was significantly enriched in the exosomes from Hotair overexpressing ADSCs (Supplementary Fig. 4a). Injection of exosomes from Hotair overexpressing ADSCs also increased Hotair expression in the intestine (Supplementary Fig. 4b), and promoted intestinal cell proliferation, as seen from both the stem cell and proliferation markers (Supplementary Fig. 4c–f). Furthermore, we loaded the human HOTAIR in the exosome (Supplementary Fig. 4g) and transferred the exosomes to HCT116. Colonosphere culture further confirmed the effects of exosomal HOTAIR (Supplementary Fig. 4h), implicating its relevance in human.

### Hotair tunes Wnt pathway in a cell context dependent manner

**Figure 6:**
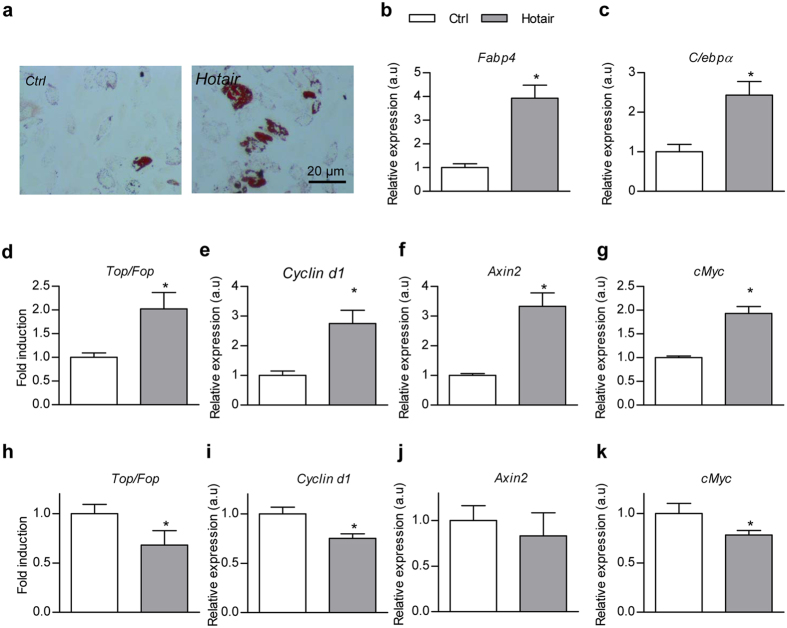
Hotair tunes Wnt pathway in a context dependent manner. (**a**) Adipogenic stem cells were infected with control or Hotair overexpressing lentivirus and followed by adipogenic induction. Lipid droplets were observed by Oil O red staining. Data presented are representative of three different experiments. (**b**,**c**) RT-qPCR analysis of the expression of adipogenic marker genes Fabp4 (**b**) and C/ebpα (**c**) in cells treated same as the above. Relative expression levels are compared with the control (arbitrary value = 1) and presented as mean 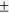 SEM (three biological replicates with three technical replicates each), *P < 0.05 as analyzed by t test. (**d–g**) Relative luciferase activity of Topflash/Fopflash reporter (**d**) and expression of Wnt downstream genes Cylin d1(**e**), Axin2 (**f**), and cMyc (**g**) in proliferating adipogenic stem cells cultured with growth medium were detected by dual luciferase assay and RT-qPCR respectively. (**h–k**) Relative luciferase activity of Topflash/Fopflash reporter (**h**) and expression of Wnt downstream genes Cylin d1 (**i**), Axin2 (**j**), and cMyc (**k**) in differentiating adipogenic stem cells cultured with adipogenic induction medium. Fold induction and relative expression levels are compared with the control (arbitrary value = 1) and presented as mean ± SEM (three biological replicates with three technical replicates each), *P < 0.05 as analyzed by t test.

Notably, Lgr5, Cyclin d1 and cMyc, all of which are Wnt pathway related genes, were all upregulated upon exosomal Hotair treatment (Fig. 5b–e and Supplementary Fig. 4c–f), suggesting that exosomal Hotair might promote intestinal cell proliferation via Wnt pathway. Previously, Hotair was found to promote adipogenesis in ADSCs^11^. The observed Wnt activation by Hotair is apparently contradict with the inhibitory role of Wnt on adipogenesis. To clarify the inconsistence, we tested the role of Hotair in ADSCs by overexpressing Hotair. In accordance with previous literature^11^, Hotair expression promotes adipogenesis under adipogenic induction, as observed from increased oil o red staining, Fabp4 and C/ebpα expression (Fig. 6a–c), together with minor inhibitory effects on Wnt pathway (Fig. 6h–k). However, in the proliferating ADSCs, Hotair overexpression increased Topflash reporter and multiple Wnt downstream targets (Fig. 6d–g), as observed in the intestine. Taken together, these data suggest a cell type and status dependent regulatory role of Hotair on Wnt pathway.

### Increased exosomal Hotair in obese subjects with sedentary lifestyle

**Figure 7:**
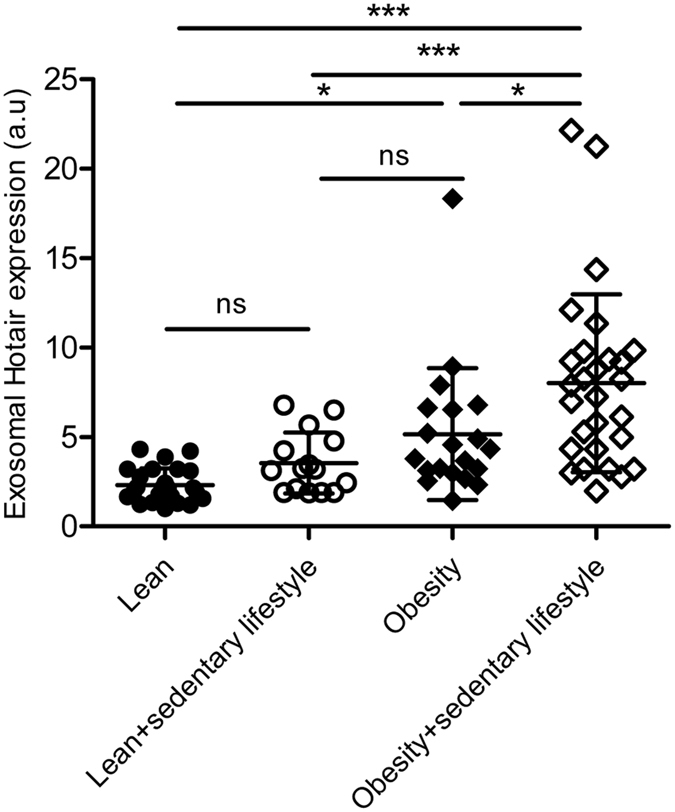
Increased exosomal HOTAIR in obese subjects with sedentary lifestyle. Eighty-nine physically healthy female subjects were recruited and divided into 4 groups based on their BMI and sedentary lifestyle: Lean (n = 26), Lean + sedentary lifestyle (n = 15), Obesity (n = 20), and Obesity + sedentary lifestyle (n = 28). Relative expression of exosomal HOTAIR was analyzed by RT-qPCR, with the lowest expression level set as value 1. *P < 0.05, ***P < 0.005 as analyzed by One way ANOVA with Tukey’s post hoc analysis.

To clarify the clinical implication and significance of the above study, 89 female subjects between 30–40 years old with variable obesity and lifestyles, were recruited for circulating exosomal HOTAIR expression analysis. All of the subjects were diagnosed without any cardiovascular and digestive diseases. They were divided into lean (BMI < 28) and obesity (BMI > 28) subgroups, which were further categorized into sedentary lifestyle or not, based on whether they were seated more than 6 hours per day. As shown in Fig. 7, there were much higher exosomal HOTAIR in the blood in obese subjects than that in lean subjects, which was further aggravated by sedentary lifestyle. Specifically, sedentary lifestyle induced a much higher circulating exosomal HOTAIR in the obese, while had minimal effects in lean subjects. All of these data suggest that obesity is a requirement for the sedentary lifestyle-induced increase in HOTAIR.

## Discussion

Although abundant epidemiological studies have established strong association of sedentary lifestyle and obesity with colorectal cancer risk, the detailed underlying mechanism remains unknown. In this study, we show that the pro-adipogenic lncRNA Hotair, which is preferentially expressed in the gluteal and femoral fat, can be secreted in the form of exosome in the context of squeeze duo to activation of NFκB. Circulating exosomal Hotair in turn can be endocytosed by intestine and thus promotes intestinal stem/progenitor cell proliferation via Wnt pathway. All of these data raise the possibility that sedentary lifestyle would affect the intestinal cell proliferation via exosomal Hotair from gluteal fat.

Recently, the reciprocal cross talk between distal cells/tissues via exosomes has been intensively studied. Exosomes from either the tumor cells or the resident cells of tumor metastasis have been found essential for metastatic tumor growth^26^,^27^,^28^,^29^. Our study here further sets an example that exosomal lncRNAs from a distal tissue/organ can circulate and be endocytosed by intestine. Moreover, our study raises the possibility that the exosomal Hotair from distal limb might contribute to the increase of Hotair in the sedentary lifestyle associated colorectal cancer patients. We found that transcriptional upregulation of Hotair, together with the increase of exosome secretion, accounts for the increased circulating exosomal Hotair upon squeeze of the gluteal-femoral fat. Exosomal RNA packaging and secretion are a fine-tuned, highly selective process and different between cancer and normal cells^25^,^30^. Further identification of cellular molecules responsible for specific Hotair encapsulation and exosome secretion is still needed, which may propose unique strategies to block the specific Hotair secretion pathway and thus helpful in preventing associated diseases.

Human HOTAIR is the first lncRNA reported to silence genes *in trans*, notably HOXD (homeobox gene cluster D) genes^6^. Additionally, mouse Hotair is also found to be involved in transcriptional repression of several imprinted gene loci^15^. Mechanistically, Hotair is thought to maintain proper gene expression levels via Polycomb- and Lsd1-related histone modification^4^. In this study, we have further revealed that Hotair could differentially regulate Wnt pathway in a cell type and stage-specific manner. The diversity of Hotair downstream effects might stem from the varied direct targets under corresponding contexts. The ability of Hotair to access to the chromatin of the target may affected by the epigenetic status of the gene loci, which is cell type and cell stage specific. Genome wide sequencing analysis followed by chromatin isolation by RNA purification^31^, would probably reveal the detailed direct target differences under these contexts. It is important to note that, besides Hotair, there might be some other effective lncRNAs or miRNAs in the gluteal-femoral fat derived exosomes functioning in the intestine. According to a recent study done by Huang *et al*., lncRNA accounted for 20.19% of the RNA in the exosome from the plasma of castration-resistant prostate cancer patients^32^. In another excellent study, lncARSR (lncRNA Activated in RCC with Sunitinib Resistance) was found encapsulated into exosomes and conferred Sunitinib resistance in renal cancer by acting as a competing endogenous RNA^25^. It is thus interesting to decode the detrimental factors in the gluteal-femoral fat derived exosomes.

Although regional fat depots function differently, there were few efficient strategies to study the differences. Homeobox genes, which encode homeodomain protein products with transcription factor characteristics, are found selectively expressed in specific somites during development. These homeobox genes are involved in the regulation of patterns of anatomical development (morphogenesis) in animals, fungi and plants^33^,^34^. In an analogous fashion, we here identified that Hotair is transcriptionally active in the gluteal-femoral fat, which is consistent with previous findings^6^,^15^. Accordingly, the site-specific expression pattern Hotair was found to promote adipogenesis in the gluteal-femoral fat depots, which is also consistent with the previous finding that HOTAIR/Hotair is an adipogenic lncRNA^11^,^17^,^35^. By analogy, it is expected that other fat depot specific Hox genes conditional KO mice would produce other regional fat defect, if it is functionally adipogenic. In fact, these Hox genes are readily available. For example, homeobox gene Lhx8 is found specifically expressed in brown fat^36^. The regional fat deficient mouse models, such as the Hotair cKO mice studied here, would provide great advantages to study the effects of regional fat on systemic diseases, especially when adipose tissues from varied depots function differently is considered.

In the clinical sample analysis, we found that higher circulating exosomal HOTAIR in the obese subjects than that in the lean subjects, which was further aggravated by sedentary lifestyle. However, sedentary lifestyle seems to have minimal effects on circulating exosomal HOTAIR in lean subjects, indicating that obesity is a requirement for the sedentary lifestyle-induced increase in HOTAIR. One explanation for this phenomenon is that the pressure load on gluteal-femoral fat in obese subjects is much higher than that in lean subjects. Another explanation might stem from the higher net mass of gluteal-femoral fat in these obese subjects, which needs to be confirmed by further correlation analysis with the regional fat mass. The net increase of gluteal-femoral fat may also explain the observed increase of exosomal HOTAIR in obese subjects. However, the latter explanation might be challenged by the widely accepted idea that gluteal-femoral fat is healthy fat^37^. As for the observed increase of exosomal HOTAIR in obese only subjects, we favor the following hypothesis. The chronic inflammation in the adipose tissue in obese subjects^38^ might activate HOTAIR expression in all the adipose depots via NFκB as observed in the gluteal-femoral fat upon squeeze, finally resulting in the increase of circulating exosomal HOTAIR. Anyway, the clinical sample analysis has implicated a possible link between circulating exosomal HOTAIR and colorectal cancer risk in obese subjects additionally with sedentary lifestyle.

Taken together, our data here set an example that exosomes carrying oncogenic RNA messages from the donor tissues could circulate into the distal receptor organs and increase its expression in the target organs. Therapeutically targeting the exosome pathway would be protective in preventing related diseases.

## Materials and Methods

### Animal husbandry

All the mice were bred in the Fourth Military Medical University Research Animal Facility and all procedures involving animal handling were in accordance with the Fourth Military Medical University guidelines. All the experimental protocols were approved by the Institutional Animal Care and Use Committee of Fourth Military Medical University. Conditional Hotair KO mice were generated similar as described before^15^. Briefly, targeting vector for Hotair was constructed by flanking the full length Hotair gene with loxP sites. For selection purpose, a neomycin resistance cassette was also inserted right after exon 2. The conditional allele was introduced by homologous recombination in iTL BA1 (C57BL/6 × 129/SvEv) hybrid embryonic stem (ES) cells by Nanjing Biomedical Research Institute of Nanjing University (Nanjing, China). The targeted ES cells were then microinjected into C57BL/6 blastocysts and the chimeras with a high percentage agouti coat color were mated to wild-type C57BL/6 mice to generate F1 heterozygous offspring. Neomycin was removed by crossed to Flper mice to get Hotair^flox/+^. Hotair KO allele was backcrossed to C57BL/6 background for at least six generations before experiments. Conditional KO mice (cKO, Hotair^flox/flox^) were crossed to FABP4-Cre transgenic mice, to yield adipose specific deletion of the Hotair locus. The heterozygous intercrosses generated Hotair wild-type (WT) and KO littermates for comparison. For genotyping, mouse tail tips were included for genomic DNA isolation. The phenotypic characterization was performed in a blinded fashion with respect to the genotypes of the animals. Mice were allowed free access to food and water and were kept at 21 °C on a 12 h-light/12 h-dark cycle.

To generate a squeeze pressure around the hindquarter mimicking the pressure load caused by sedentary lifestyle and to reduce the physical activity, we have designed the following experimental procedure for the first time. Briefly, mice were forced to wear a customized pneumatic cuff (6 mm width) around the most proximal portion of both thighs. The air pressure for the pneumatic cuffs was set at 20 mmHg. In addition, the gluteal region surround the tail was also clamped with a clip for 1 hour every night. The squeeze was conducted for 3 weeks before following analysis. Mice used for intestinal function analysis were about 6 months old.

### Whole embryo and tissue section *in situ* hybridization

About 400 bp Hotair cDNA was cloned into pGEM-T vector (Promega) and sequenced for confirmation of the cloning direction (with primers shown in Supplementary Table 1). Following linearization with Nde1 and Nco1 respectively, antisense and sense control RNA probes were synthesized by *in vitro* transcription with T7 or Sp6 RNA polymerase (MAXIscript^®^ T7/Sp6 *In Vitro* Transcription Kit, Ambion), per manufacturer instructions using Digoxigenin (DIG)-11-UTP (Roche, Germany). Whole mount *in situ* hybridization of E16.5 embryos and *in situ* hybridization on tissue sections were performed per prior methods^39^. Single-molecule *in situ* hybridization was performed using NBT/BCIP from DIG Nucleic Acid Detection Kit (Roche).

### Exosome isolation and characterization

Exosomes isolation from the adipose tissue was done following a previously published protocol^13^,^14^. Briefly, the gluteal adipose tissue from both control and experimental mice were washed with PBS and cut into 4 mm^3^ pieces, transferred to 6-well plates containing 3 ml/well of Dulbecco’s modified Eagles medium (Invitrogen, Carlsbad, CA) supplemented with 50 μg/ml gentamicin. The culture supernatant was centrifuged at 5,000 g for 15 min to remove cells and debris. Exosomes were then isolated from cultured supernatants using ExoQuick-TC Precipitation Solution (System Biosciences, Mountain View, CA). The exosomes were diluted to 10 μg/ml with Milli-Q water for size determination using a Nano ZS (Malvern Instruments, Worcestershire, UK). To visualize exosomes *in situ*, the adipose samples were cut as small as possible before fixed with 4% Glutaraldehyde in sodium cacodylate buffer on ice (+4 °C), followed by post-fixture in 2% osmium tetroxide. After dehydration in an ethanol series, the sample was embedded in Glycid ether 100 (Epon 812 equivalent). After polymerization, ultrathin sections were cut using a diamond knife and contrasted by Reynolds method for routine transmission electron microscopy (TEM). Ultrastructural investigations were performed using a transmission electron microscope (JEM1230, JEDL, Tokyo, Japan) operated at 80 kV.

Isolated exosome samples were also validated by transmission electron microscope (TEM). The sample (10 μL) was placed on the grids and allowed to stand at room temperature for 90 sec. Excess of fluid was removed by touching the edge with filter paper. Samples were then examined after negative staining with phosphotungstanic acid before subjected to observation under a transmission electron microscope (JEM1230, JEDL, Tokyo, Japan) at an acceleration voltage of 20 kV.

For exosomal RNA detection, the cultured medium for exosome isolation were first treated with RNase (Qiagen, final concentration: 5 μg/mL) at 37 °C for 30 min and then subjected to proteinase K (Tiangen, final concentration: 100 μg/mL) at 50 °C for additional 30 min. After the treatment, exosomes were isolated as described above for the following RNA expression detection using RT-qPCR.

### Plasmid construction and virus package

The cDNA sequence of mouse Hotair and human HOTAIR without polyA site were cloned into pWPI vector using the PacI and BstB1 enzyme sites (Plasmid #12254, Addgene). Control pWPI or pWPI-Hotair (6.25 μg), pMD2.G (Plasmid #12259, Addgene) (0.625 μg) and psPAX2 (Plasmid #12260, Addgene) (3.125 μg) vectors were co-transfected into 80% confluent 293T cells using Lipofectamine2000 per manufacturer’s protocol (Invitrogen). Virus supernatant was then harvested 2 days after transfection and then filtered with 0.45 μm filter. Adipose derived mesenchymal stem cells from the indicated fat depots were isolated and cultured as described before^40^. Cells were allowed to grow to 30–50% confluence before infected with the above lentivirus with the help of 8 μg/mL polybrene (Santa Cruz Biotechnology).

### Exosome labeling, loading of Hotair, and *in vivo* injection

For *in vivo* tracking exosomes, purified exosomes were labelled with fluorescent dye DiI at the final concentration of 8 μM (Invitrogen) at RT for about 30 minutes. Then, labelled exosomes were washed with PBS, followed by ultracentrifugation and resuspension in PBS before use. Mice were then received 10 μg of exosomes (protein concentration) dispersed in 200 μL PBS via tail vein injection. Tissues from interested organs were harvested 2 hours post injection and then fixed using O.C.T in the mold at −80 °C and cut into 8 μm slices. Tissue cryo-sections were fixed for 15 mins by 4% paraformaldehyde and counterstained with Hoechst (Invitrogen). The red fluorescence for the labeled exosomes and the blue nuclei were viewed by laser scanning confocal microscope (ECLIPSE Ti, Nikon, Tokyo, Japan).

For loading mouse Hotair/human HOTAIR RNAs into exosomes, cultured ADSCs were infected with pWPI control or pWPI-Hotair/HOTAIR. Exosomes from the culture medium of the control and Hotair/HOTAIR overexpressed cells were purified in a similar procedure as described above. Loading of Hotair/HOTAIR was confirmed by RT-qPCR.

### Immunostaining

As previously described^1^, freshly isolated intestinal tissues were rinsed with PBS, and excessive fluid was removed by touching the edge with filter paper. Then the trimmed tissues were OCT embedded and sectioned. For immunostaining of nuclear translocation of p65, rabbit anti-p65 NFκB (1:200, Sc-109, Santa Cruz) was used. The sections were made permeable with 0.5% Triton X-100 in PBS, rinsed with 100 mM glycine in PBS, blocked with 10% donkey serum in PBS, incubated overnight with primary antibody at 4 °C. Immunodetection was performed in a 3-step protocol, using streptavidin-horseradish peroxidase complex, with visualization by 3,3-diaminobenzidine.

### Adipogenic Differentiation Induction

For adipogenic induction, ASCs cultured in the growth medium were converted into adipogenic induction medium (50 *µ*M 3-isobutyl-1-methylxanthine (Sigma-Aldrich), 10 *µ*M dexamethasone (Sigma-Aldrich), 10 *µ*M rosiglitazone (Sigma-Aldrich), and 10 *µ*g/mL insulin) one day post confluence. The medium was changed every 3 days. The differentiated cells were fixed in 10% formaldehyde for 15 minutes at room temperature and lipid droplets were identified by Oil red O (Sigma-Aldrich) staining.

### Colonosphere culture

Three-dimensional multicellular spheroids (Colonosphere) were cultured as previously described^41^,^42^. The human CRC cell line HCT116, was originally purchased from the American Type Culture Collection (ATCC) (Cat#ATCC-CCL-247). The cells were cultured in stem cell medium (Dulbecco’s modified Eagle medium/F12; Gibco) with 0.6% glucose (Sigma, Germany), 2 mM l-glutamine, B27 (Invitrogen), 10 mL antibiotic-antimycotic, 4 μg/mL gentamicine, 20 ng/mL epidermal growth factor (R&D, USA), 100 μM β-mercaptoethanol (Sigma, Germany), and 10 ng/mL basic fibroblast growth factor (R&D, USA), in nonadherent culture flasks (Corning, USA) at 37 °C in a 5% CO2-humidified incubator. The culture media was changed every second or third day. Spheres were quantified on day 7 unless otherwise specified.

### RT-qPCR

Tissues, cells or exosomes were lysed in Trizol Reagent (Invitrogen) and total RNA was isolated according to the manufacturer’s instructions with following modification for exosomal RNA purification: the aqueous phase containing total RNA was purified using the RNeasy plus kit (Qiagen). RNA was converted to cDNA with the cDNA synthesis kit (M-MLV, Promega). RT-qPCR was performed with diluted cDNA (1:10) in three wells for each primer and SYBR green master mix (Bio-Rad) on Bio-Rad iCycler RT-PCR detection system. All RT-qPCR experiments were repeated at least three independent times. Relative expression was calculated with 2^−ΔΔCt.^, using β-actin as a reference gene and compared with the indicated control sample(s). Housekeeping gene β-actin was also used as a reference gene for exosomal lncRNAs according to previous literatures^43^,^44^,^45^, for the following reasons. On one hand, lncRNAs, specifically Hotair are RNAs with polyA end, and thus only the RNAs with polyA are suitable for reference. On the other hand, it seems that the genes abundantly expressed in cells have the trend to be present at levels comparable to its cellular levels. Primers used are listed on Supplementary Table 1.

### ChIP

Proliferating adipogenic stem cells were treated with TNFα for 6 hours, followed by formaldehyde cross-linking. Alternatively, the squeezed gluteal fat were cut to small pieces and followed by formaldehyde cross-linking. Immunoprecipitation was performed with control IgG or p65 antibody. The nuclei were lysed in lysis buffer (50 mM Tris-HCl (pH = 8), 10 mM EDTA, 1% sodium dodecyl sulfate (SDS), 1 mM phenylmethylsulfonyl fluoride, protease inhibitors) and sonicated on ice to obtain 250 to 800 bp DNA fragments. After preclearing for 1 h and incubation with antibodies overnight, the p65 interacting DNA was precipitated by Protein A beads according to the manual instruction. After reversal of formaldehyde crosslinking, the immunoprecipitated complex was treated with RNase A and proteinase K before DNA purification for PCR analysis (Supplementary Table 1).

### Western Blot

Gluteal fat or the isolated exosomes were lysed in lysis buffer. Proteins were separated by SDS–PAGE electrophoresis before transferred onto a nitrocellulose membrane. After blocking with 5% milk for 30 min, the membranes were incubated with various primary antibodies (anti-GM130, anti-Alix, anti-CD9, and anti-GAPDH respectively) overnight at 4 °C, followed by incubation with secondary antibodies for 1 h at room temperature, and visualized with enhanced chemiluminescence reagent (Thermo Scientific).

### Flow cytometry

The human CRC cell line HCT116 treated with indicated exosomes were harvested and fixed for at least 2 h in PBS containing 70% ethanol. Cells were then spun down gently in 200 μl extraction buffer (0.1% Triton X-100, 45 mM Na2HPO4 and 2.5 mM sodium citrate) at room temperature for 20 min and then resuspended in PBS containing 40 mg/ml PI, 0.1 mg/ml RNase (Qiagen) and 0.1% Triton X-100 for 30 min in dark. Cell cycle distribution was detected by FACS (Becton-Dickinson San Jose, CA).

### Study Population and clinical sample collection

Eighty-nine eligible female participants aged between 30 and 40 years at enrollment were recruited and accepted to give written informed consent and completed questionnaires on their lifestyle, and medical history. Subjects were then invited to hospital to provide a blood sample and to have anthropometric measurements taken. Approval for this study was obtained from the ethical review boards of the Fourth Military Medical University. All the procedures were performed in accordance with the Clinical Sample Collection and Preparation Guidelines of the Fourth Military Medical University.

### Statistics

All experiments reported were repeated at least three independent times. All the data were first subjected for normality test, followed by statistical analysis. Data are expressed as mean ± SEM. For statistical significance of the differences between the means of two groups, we used two-tailed Student’s t-tests. One way ANOVA with Tukey’s post hoc test is used for multiple comparisons. No samples or animals were excluded from analysis, and sample size estimates were not used. Animals were randomly assigned to groups. Significance is considered at p < 0.05.

## Additional Information

**How to cite this article:** Lu, X. *et al*. Sedentary lifestyle related exosomal release of Hotair from gluteal-femoral fat promotes intestinal cell proliferation. *Sci. Rep.* **7**, 45648; doi: 10.1038/srep45648 (2017).

**Publisher's note:** Springer Nature remains neutral with regard to jurisdictional claims in published maps and institutional affiliations.

## Supplementary Material

Supplementary Information
