# Supplementary material for: Sedentary lifestyle related exosomal release of Hotair from gluteal-femoral fat promotes intestinal cell proliferation
Source: Sci Rep. 2017 Mar 31;7:45648. doi: 10.1038/srep45648 (PMC5374500; doi:10.1038/srep45648)
Supplement: Supplementary Information [file srep45648-s1.pdf]

Supplementary Data

Sedentary lifestyle related exosomal release of Hotair from gluteal-femoral fat promotes intestinal cell proliferation

Running title: Exosomal Hotair promotes intestinal proliferation

Xiaozhao Lu[1,2,3#], Danna Bai[3,4#], Xiangwei Liu[1,2,5#], Chen Zhou[6], Guodong Yang[1,2*]

[1] Department of Biochemistry and Molecular Biology, Fourth Military Medical University, Xi'an, 710032, China

[2] State Key Laboratory of Cancer Biology, Fourth Military Medical University, Xi'an, 710032, China

[3] The 323rd Hospital, PLA, Xi'an, 710043, China

[4] Department of Physiology, Fourth Military Medical University, Xi'an, 710032, China

[5] State Key Laboratory of Military Stomatology and National Clinical Research Center for Oral Diseases, Fourth Military Medical University, Xi'an, 710032, China

[6] Guanghua School of Stomatology, Hospital of Stomatology, Guangdong Provincial Key Laboratory of Stomatology, Sun Yat-sen University, 56 Lingyuanxi Road, Guangzhou, 510055, China

[*]Correspondence should be addressed to Guodong Yang (yanggd@fmmu.edu.cn).

[#] Xiaozhao Lu, Danna Bai, and Xiangwei Liu contribute equally to this study.

Supplementary Table1 Primers/sequences used in this study.

Primer name	Forward	Reverse
Human HOTAIR qPCR	5'CAAACAGAGTCCGTTCAGTGTCA3'	5'TAATTCTTAAATTGGGCTGGGTC3'
Human ACTB qPCR	5'GCACAGAGCCTCGCCTT3'	5'GTTGTCGACGACGAGCG3'
Mouse Hotair qPCR	5'GCTCGCCCCTTGAAACCCTCTT3'	5'GCGTGGTCAGATCGCTGGTCAT3'
Mouse Actb qPCR	5'TTCTTTGCAGCTCCTTCGTT3'	5'ATGGAGGGGAATACAGCCC3'
Mouse Hotair in situ	5'CCACGCACATCTATCTCCACCG3'	5'CTTCTCCCAGGAAGGGCAAAGG3'
Human HOTAIR cloning	5'GGGTTTAAACACTCGCCTGTGCTCTGGAGCTTGATC3'	5'GGTTCGAAGAAAATGCATCCAGATATTAATATATCT3'
Mouse Hotair cloning	5'GGGTTTAAACAAAACTGTAATACTCAGACAGACA3'	5'GGTTCGAACACGTGTATCTACATTCTGTAGC3'
Musashi1	5'GGTTTCGGCTTCGTCACTTTCAT3'	5'ACCATCTTAGGCTGTGCTCTTCG3'
Axin2	5' ACTGACCGACGATTCCATGT3'	5' TGCATCTCTCTCTGGAGCTG3'
Lgr5	5'AGCTACCCGCCAGTCTCCTACAT3'	5'CATAAGCACTTTGAGGCTGTGAA3'
Rab27b	5'AGGAAGAAACAAGGTAAAACAG3'	5'CAGATAGAAAAGGAAGCAGAAA3'
Cyclind1	5'TCCTCTCCAAAATGCCAGAG3'	5'GGGTGGGTTGGAAATGAAC3'
cMyc	5'AGAGCTCCTCGAGCTGTTTG3'	5'TGAAGTTCACGTTGAGGGG3'
Alix	5'ATTCAAGGACCGCTGGCAAAGGA3'	5'GCGATGGTGTCTCGATGGGACTG3'
Fabp4	5'ACAAGCTGGTGGTGGAATGTG3'	5'CCTTTGGCTCATGCCCTTT3'
C/ebpα	5'TGCGCAAGAGCCGAGATAAA3'	5'CCTTCTGTTGCGTCTCCACG3'
MouseHotairChIP1	5'CAACCATTTGGAATGCTGTG	5'TGAGGGTAGCCAAGGTAGGG

	AGC3'	ACA3'
MouseHotairChIP2	5'AGCCCACTTTGGGGATCTTATAG3'	5'TTGTTGTTACCTTTGCCTTGTCA3'
MouseHotairChIP3	5’ AACCAAGAACGACCTAAT3’	5'GAGGTGGAAAGGAAAGAAC3'

Supplementary Figures

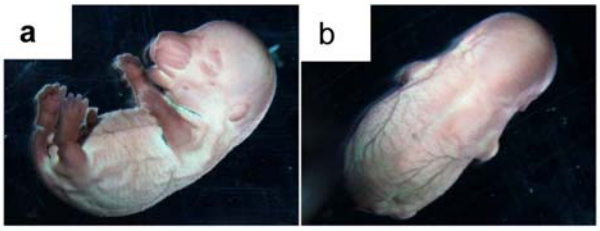

Supplementary Figure S1 Hotair expression in E16.5 embryo.

a,b, Whole mount in situ hybridization of Hotair in E16.5 mouse embryo. Data presented are representative of three embryos.

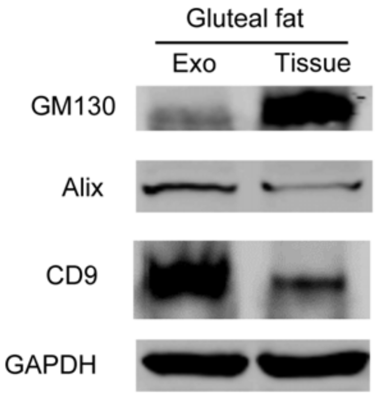

Supplementary Figure S2 Western blot characterization of exosomes.

Gluteal fat derived exosomes or the parental tissue lysis were subjected for Western blot analysis of the expression of GM130, Alix, CD9 and GAPDH. Data presented are representative of three different experiments.

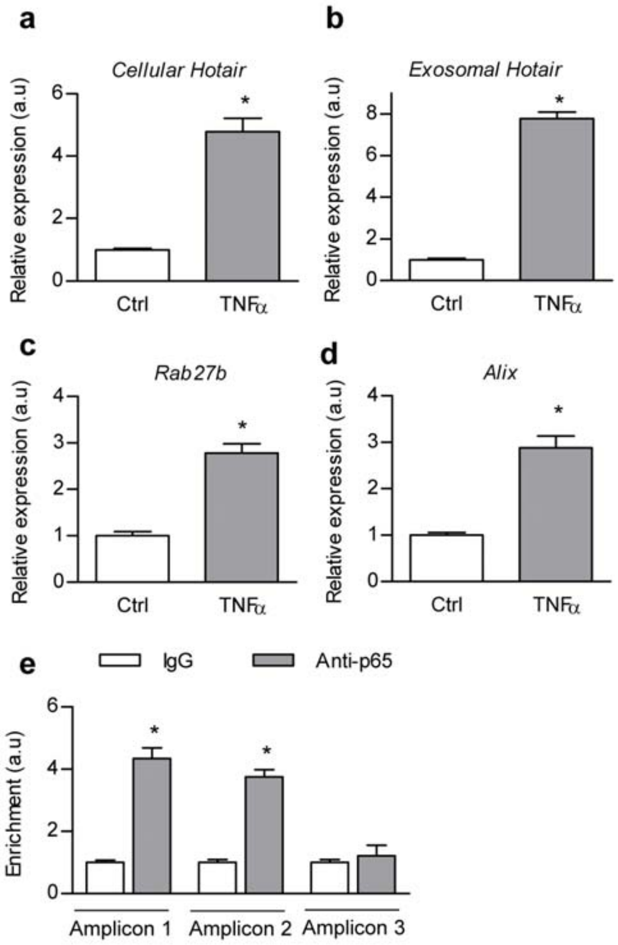

Supplementary Figure S3 TNFα promotes Hotair expression in ADSCs via p65.

a, b, RT-qPCR analysis of Hotair expression in control and TNFα treated ADSCs (**a**) and exosomes (**b**) from the above cells. **c, d,** RT-qPCR analysis of exosome biogenesis related Rab27b (**c**) and Alix (**d**) in control and TNFα treated ADSCs. Data are compared with the control (arbitrary value=1) and presented as mean± SEM (three biological replicates with three technical replicates each), *, $P<0.05$ by t test. **e,** Chromatin Immunopreciptation (ChIP) analysis of the putative interaction between p65 and the predicted cis-elements on Hotair promoter in ADSCs treated with 10 ng/ml TNFα. Data are compared with the IgG control (arbitrary value=1) and presented as mean± SEM (three biological replicates with three technical replicates each), * denotes $P<0.05$ by One Way ANOVA with Tukey's post hoc test.

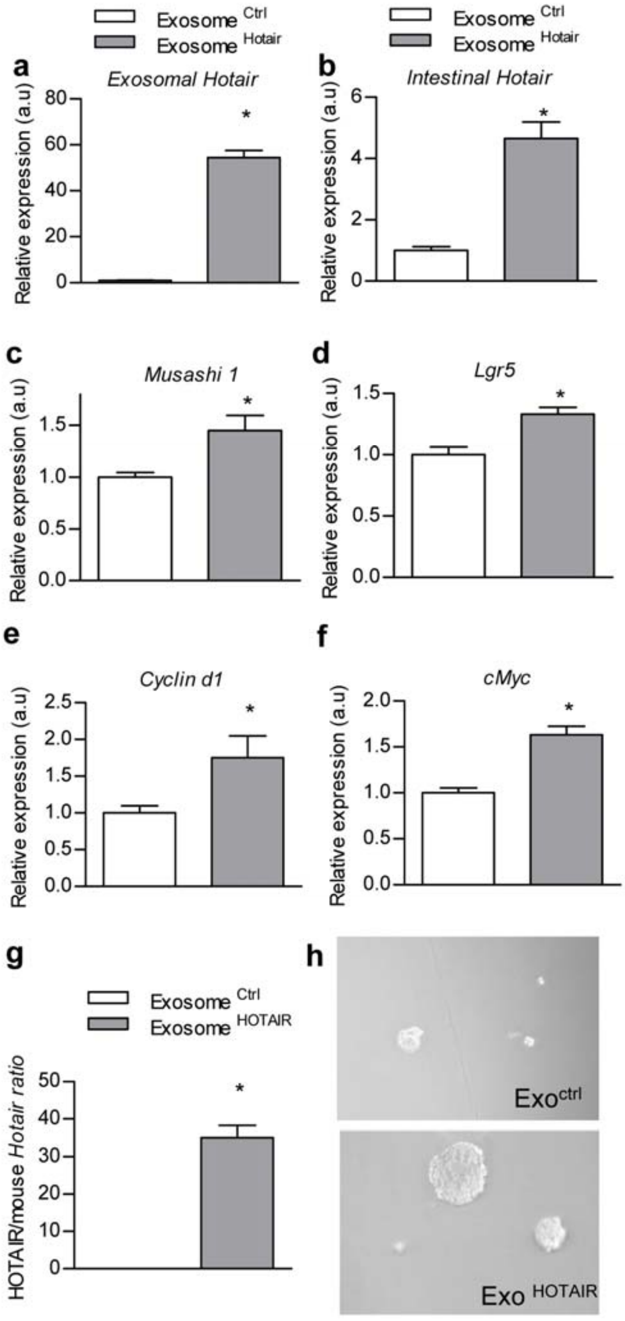

Supplementary Figure S4 Exosomal Hotair promotes intestinal cell proliferation.

a, RT-qPCR analysis of Hotair expression in the exosomes derived from control or Hotair transfected ADSCs. **b-f,** Mice were treated with exosomes encapsulated with or without Hotair and intestinal expression of Hotair (**b**), Musashi1 (**c**), Lgr5 (**d**), Cyclin d1 (**e**), and cMyc (**f**) was analyzed by RT-qPCR. Data are compared with the control (arbitrary

value=1) and presented as mean±SEM (three biological replicates with three technical replicates each), *P<0.05 as analyzed by t test. **g,** Exosome from HOTAIR transfected ADSCs were harvested and relative expression of human HOTAIR was expressed as human HOTAIR/mouse Hotair ratio using RT-qPCR . Data are presented as mean±SEM (three biological replicates with three technical replicates each), *P<0.05 as analyzed by t test. **h,** Colon cancer cells were incubated with exosomes with or without HOTAIR delivery and colonospheres were observed under light microscope. Data presented are representative of five experiments.
